# Evaluation of the incidence, predictors, risk assessment scores and outcomes of thromboembolism in a cohort of Egyptian NHL patients - Real World Experience

**DOI:** 10.1007/s00277-024-05904-8

**Published:** 2024-08-07

**Authors:** Shaimaa El-Ashwah, Salma Elashwah, Omnia Khaled, Ahmed A. Ghanem, Hadeer Elsaeed AboElfarh, Ramadan Ayman Selim, Reham Osama Mansour, Yasmine Shaaban

**Affiliations:** 1 Hematology Unit, Internal Medicine Department, Oncology Center, Mansoura University, Mansoura, Egypt; 2 Medical Oncology Unit, Internal Medicine Department, Oncology Center, Mansoura University, Mansoura, Egypt; 3 Hematology Unit, Clinical Pathology Department, Mansoura University, Mansoura, Egypt; 4 Manchester Program, Faculty of Medicine, Mansoura University, Mansoura, Egypt; 5 Neurology Department, Faculty of Medicine, Mansoura University, Mansoura, Egypt; 6 Internal Medicine Department, Faculty of Medicine, Mansoura University, Mansoura, Egypt

**Keywords:** VTE, Predictors, ANC, NLR, PLR

## Abstract

Non-Hodgkin’s Lymphoma (NHL) is the most common subtype of lymphoma. The incidence of venous thromboembolism (VTE) in aggressive NHL was estimated recently to be 11%. Several risk assessment scores and factors are available to help identify cancer patients at risk for developing VTE. Patients with a pathologically confirmed diagnosis of NHL were identified at the Oncology Center of Mansoura University. The study included 777 patients: 719 with DLBCL-NOS, 26 with Anaplastic-B-cell, and 32 with T-cell-rich-NHL. Data were retrospectively collected from electronic medical records, including clinical, radiological, and laboratory information related to VTE and NHL. The median age at NHL diagnosis was 53 years, (range: 18–98). There was a male predominance, 51.4% of the cases. At initial lymphoma diagnosis, VTE was identified in 46 (5.9%) patients, and 61 (7.9%) patients experienced VTE while undergoing chemotherapy. According to logistic regression analysis, a PS (performance status) ≥ 2, bulky lesions, and mediastinal masses were significant predictors of VTE at presentation, with *P*-values of 0.022, 0.002, and < 0.001, respectively. Meanwhile, NHL patients who developed VTE during chemotherapy had significantly poorer PS, higher absolute neutrophilic counts (ANC), neutrophil/lymphocyte ratio (NLR), platelet-lymphocyte ratio (PLR) and lactate dehydrogenase (LDH) levels than lymphoma patients without VTE, with *P*-values of 0.003,  0.034, 0.049, 0.01 and 0.007, respectively, as determined by multivariate analysis. The ROC curve identified the cut-off values of 4.875 × 10^9^/L for ANC, 2.985 for NLR, 144.85 for PLR, and 417.5 U/L for LDH as potential markers for predicting VTE in NHL patients. Patients with a PS ≥ 2 and values exceeding these cut-offs for ANC, NLR, and PLR experienced significantly higher incidences of VTE than other groups, with *P-*values of 0.003, < 0.001, < 0.001, and < 0.001, respectively. At the end of the follow-up, the overall survival was significantly shortened by VTE occurring during chemotherapy, hypoalbuminemia,  intermediate-high and high international prognostic index (IPI) scores (intermediate-high and high), responses other than CR and relapse, all with *P-*values < 0.05. ECOG PS and Inflammatory markers such as NLR, PLR, and neutrophilic count could serve as predictors of the development of thrombotic events in patients with NHL-DLBCL. Additionally, the occurrence of VTE during chemotherapy is an independent poor prognostic marker for overall survival (OS).

## Introduction

Non-Hodgkin’s Lymphoma (NHL) is the most commonly diagnosed subtype of lymphoma [1]. In 2023, it was estimated that there were 80,550 new cases of NHL in the United States [2]. In Egypt, NHL accounted for 5.4% of all cancer cases in 2020 [3]. Among the subtypes of NHL, diffuse large B-cell lymphoma (DLBCL) is considered the most aggressive [4]. The occurrence of venous thromboembolism (VTE) in patients with malignancy is well-documented, and researchers continue to investigate the pathophysiology and risk factors associated with this condition [5]. VTE in patients with malignancy contributes to higher mortality rates, prolonged hospital stays, and increased treatment costs [6]. Lymphomas present the highest risk for cancer-associated thrombosis (CAT) among hematologic malignancies [7]. The incidence of VTE in aggressive NHL was recently estimated to be 11%, ranking it second only to acute lymphoblastic leukemia among hematological malignancies in terms of VTE incidence [8].

Risk factors for cancer-associated thrombosis (CAT) beyond malignancy type include the use of chemotherapies such as anthracyclines and cisplatin, which are commonly used in lymphoma treatment protocols and induce a higher risk of VTE in lymphoma patients [9–11]. Other factors include immunotherapies, inherited thrombophilia, a history of thrombosis, obesity, advanced age, and susceptibility to infections [12].

Blood counts measured before the initiation of chemotherapy are considered risk factors for CAT. Notable factors include a white blood cell (WBC) count of 11 × 10^9^/L or higher, a hemoglobin concentration below 10 g/dL, and a platelet count of 350 × 10^9^/L or higher [11, 13]. Lymphomas induce an inflammatory state characterized by immune dysregulation and the release of pro-inflammatory cytokines, such as interleukin-6 (IL-6), IL-10, and tumor necrosis factor-alpha (TNF-α), which are associated with the development of NHL [14]. While WBC count has traditionally been used as a biomarker of inflammation, newer biomarkers such as the neutrophil-to-lymphocyte ratio (NLR) and platelet-to-lymphocyte ratio (PLR) are now being widely evaluated to assess their roles in hematological malignancies and their potential as predictors of CAT [15]. Additionally, some studies have found that a low mean platelet volume (MPV), which is both a marker of platelet activity and an inflammatory biomarker, was associated with the development of VTE in lymphoma patients [16–18].

The Eastern Cooperative Oncology Group performance status (ECOG PS) of 2 or higher is a well-recognized poor prognostic factor for lymphoma and its overall survival (OS). It is also considered a risk factor for developing VTE in these patients, although the specific ECOG PS score associated with VTE risk can vary across different studies. Additionally, elevated levels of lactate dehydrogenase (LDH), one of the variables in the international prognostic index (IPI), was also considered as a risk factor for VTE in lymphoma in various studies [19].

Several risk assessment scores are available to help identify cancer patients at risk for developing VTE. In the Khorana risk stratification model, the rates of symptomatic VTE in the low, intermediate, and high-risk groups were 0.3%, 2%, and 6.7%, respectively, over a median follow-up period of 2.5 months [13]. The Thrombosis Lymphoma (ThroLy) risk assessment model (RAM) offers a more specific score for estimating the risk of VTE in lymphoma patients and is noted for its high predictive value [20]. In the CONKO-score, the variables of the Khorana score were utilized, with the substitution of body mass index (BMI) for the World Health Organization (WHO) PS [21].

In this retrospective study, we aimed to estimate the incidence of VTE in B-cell NHL, assess the clinical and laboratory status of these patients, identify risk factors for developing VTE, evaluate the effectiveness of thrombosis risk assessment models in predicting VTE in NHL patients, and lastly evaluate the clinical impact of VTE on patient outcomes.

## Patients and methods

This retrospective study included 777 NHL patients who were diagnosed, and treated at Oncology Center Mansoura University (OCMU), Egypt, from January 2009 to 2021. Patients with pathologically confirmed diagnosis of NHL [including DLBCL-NOS (*n* = 719), Anaplastic-B-cell (*n* = 26), and T-cell-rich-NHL (*n* = 32)] were identified and data were extracted from Oncology Center Mansoura University electronic medical records.

VTE cases were radiologically confirmed; deep vein thrombosis (DVT) was diagnosed by Doppler ultrasound and computed tomography (CT) angiography was used to diagnose pulmonary embolism (PE) for symptomatic cases; routine screening for VTE was not done. Thrombosis at presentation was documented according to the patient’s history records and archived radiological data and thrombosis was considered chemotherapy-related if occurred at any time after the first chemotherapy cycle. Clinical, radiological and laboratory data related to VTE, including patients’ age, gender, comorbidities, ECOG PS, personal history of VTE, virology status, liver cirrhosis, B symptoms, lymphoma burden, IPI score, LDH levels, complete blood count (CBC) parameters including white blood cell, differential leucocytic (DLC) and platelet counts, Hb levels, red blood cells and platelet indices, liver, and renal function tests were collected and documented.

Variables in the **Khorana** (primary tumor site, platelet count [≥ 350 × 10^9^/L], Hb concentration [< 10 g/dL], WBC count [≥ 11 × 10^9^/L], and a BMI[≥ 35 kg/m^2^]), **CONKO** (the same variables in Khorana score with WHO ECOG PS substituting BMI) and **ThroLy** (previous venous and/or arterial thromboembolic events, mediastinal involvement, obesity with BMI [> 30 kg/m^2^], Hb concentration [< 10 g/dL], extranodal localization, reduced mobility, and neutropenia [absolute neutrophil count (ANC) < 1 × 10^9^/L]), and scores were calculated for 311, 777, and 311 patients, respectively.

Most patients were treated by the following first line protocols with or without rituximab (R);CHOP (Cyclophosphamide, Doxorubicin, Vincristine, and Prednisone), others received Dose-adjusted etoposide, prednisone, vincristine, cyclophosphamide, and doxorubicin (DA-EPOCH), while elderly or frail patients with comorbidities received mini-CHOP or COP. Patients who did not achieve complete remission (CR) received salvage therapy [DHAP, ICE, MINE, or gemzar-based protocols] after fitness reassessment. Radiotherapy consolidation for patients with bulky tumors at presentation or residual lymphadenopathy. Response evaluation to first-line and salvage chemotherapies was collected according to CT and/or PET-CT scans and clinical follow-up data [22, 23].

### Statistical analysis

Data were analyzed using the Statistical Package of Social Science (SPSS) program for Windows (Standard version 21). The normality of data was first assessed with a one-sample Kolmogorov-Smirnov test. Qualitative data were described using numbers and percentages and compared using the Chi-square test. Continuous variables were presented as median (min-max) for non-normal distributed data. For comparing two groups Mann Whitney test (non-parametric) was used. The ROC Curve (receiver operating characteristic) was used to assess the sensitivity and specificity for quantitative diagnostic measures that categorize cases into one of two groups. Kaplan- Meier test was used for survival analysis and the statistical significance of differences among curves was determined by the Log-Rank test. Binary logistic regression was used to evaluate factors affecting VTE at presentation. Cox’s proportional hazards regression model was used to evaluate factors affecting both VTE at chemotherapy and overall survival. For all the above-mentioned statistical tests done, the results were considered significant when the *p* ≤ 0.05. The smaller the p-value obtained, the more significant the results.

## Results

### Patients’ and lymphoma characteristics

Median age was 53 years (range: 18–98) with 29.2% having advanced age (> 60 years) at diagnosis, there was male predominance (51.4%). At presentation, 157 (20.2%) patients had an ECOG PS of 2–4. Two-hundred and fifty-four patients (32.7%) had B symptoms at presentation. Advanced disease (Ann Arbor Stage III/IV), at diagnosis, was documented in 588 (75.7%) of lymphoma patients. Bulky disease (tumor size > 10 cm), mediastinal involvement, and more than 1 extranodal site involvement were illustrated in 240 (30.9%), 32 (4.1%), and 126 (16.2%) patients, respectively. From the available data, we calculated the IPI for 703 patients and they were divided into 4 risk categories: low risk (*n* = 167), low-intermediate risk (*n* = 266), high-intermediate (*n* = 189), and high (*n* = 81). Anthracycline-based chemotherapy ± Rituximab was the first-line treatment administrated in 92.8% of the study population. 457 (58.8%) patients achieved complete response (CR) after 1^st^ line treatment. One hundred and nine (23.9%) patients relapsed after CR. At the end of the study follow up 446 (57.4%) were alive while 150 (19.3%) died and 181 (23.3%) had lost follow-up Table 1.

**Table 1:** Patients’ characteristics among the studied group

Patients’ characteristics		The studied group (*n* = 777)
Age (years)	Median (Min-Max)	53.0 (18–98)
Gender	Male Female	399 (51.4%) 378 (48.6%)
Comorbidities	Negative HTN DM HTN/DM Unknown	533 (68.6%) 69 (8.9%) 77 (9.9%) 46 (5.9%) 52 (6.7%)
Virology	Negative HCV HBV Unknown	321 (41.3%) 333 (42.9%) 10 (1.3%) 113 (14.5%)
PS	< 2 2–4	620 (79.8%) 157 (20.2%)
IPI	Low Low-intermediate Intermediate-high High Unknown	167 (21.5%) 266 (34.2%) 189 (24.3%) 81 (10.4%) 74 (9.5%)
Hospital stay	No Yes	668 (86.0%) 109 (14.0%)
Khorana risk score (*n* = 311)	Intermediate High	262 (84.2%) 49 (15.8%)
CONKO risk score	Low High	649 (83.5%) 128 (16.5%)
Throly risk score (*n* = 311)	Low Intermediate High	121 (38.9%) 160 (51.5%) 30 (9.6%)
Chemotherapy type	Non-anthracycline-based Anthracycline based	56(7.2%) 721(92.8%)
Overall response	Yes (CR + PR) No (SD + PD) Not assessed	613 (78.9%) 148 (19%) 16 (2.1%)
Relapse (*n* = 457)	Non relapsed Relapsed	348 (76.1%) 109 (23.9%)
Thrombosis at presentation	No Yes	731 (94.1%) 46 (5.9%)
Thrombosis on chemotherapy	No Yes	716 (92.1%) 61 (7.9%)
Time to thrombosis – (months)	Median (Min-Max)	8.0 (1.0–96.0)
Living status	Alive Dead Lost follow up	446 (57.4%) 150 (19.3%) 181 (23.3%)

### Venous thromboembolism (VTE)

**Fig. 1:**
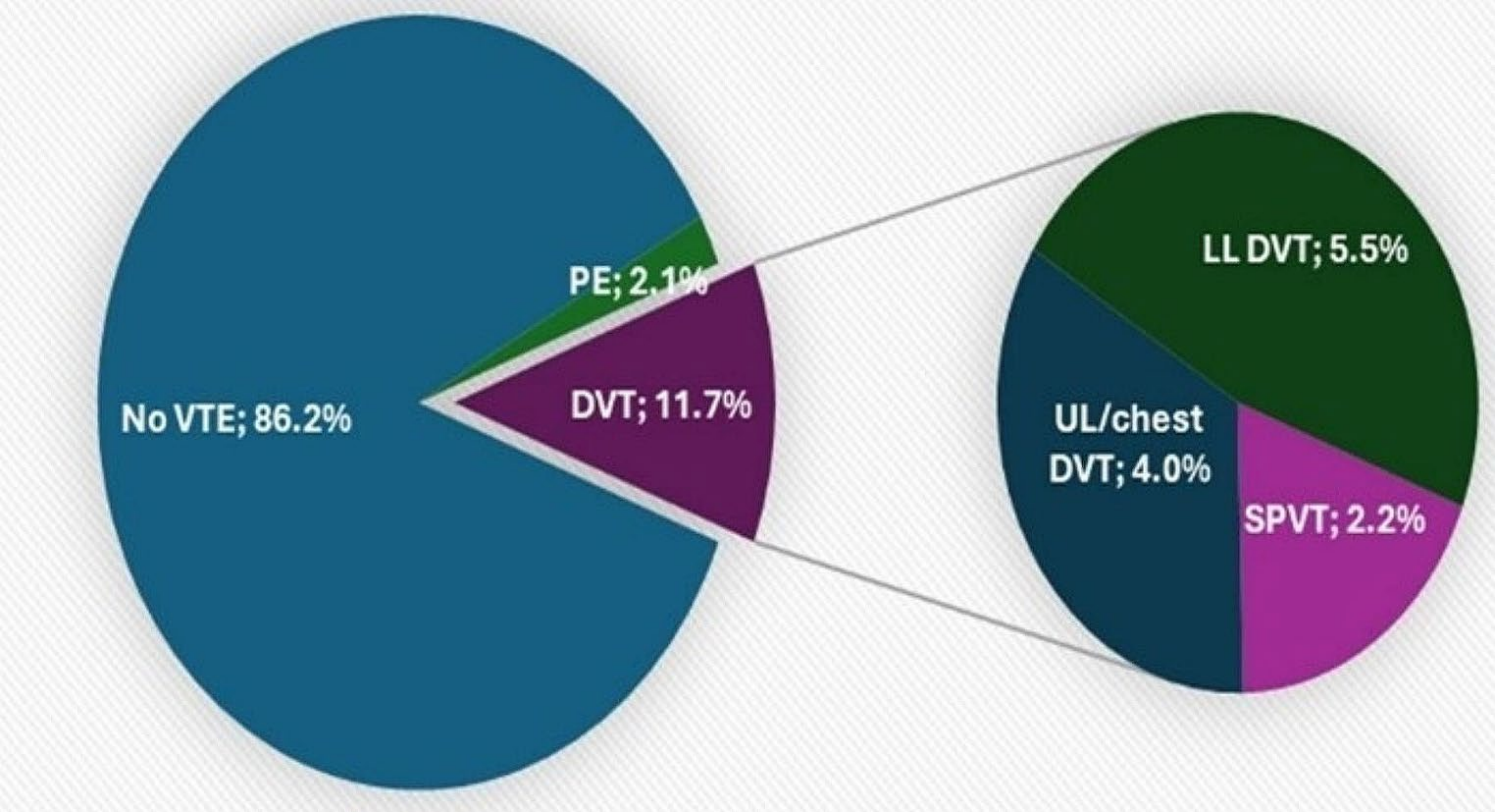
Types and frequencies of detected VTE. VTE: venous thromboembolism, PE: Pulmonary embolism, DVT: deep venous thrombosis, UL/chest DVT: upper limb/ chest dep venous thrombosis, LL DVT: Lower limb deep venous thrombosis, SPVT: Splanchnic Vein Thrombosis

VTE was reported in 107 (13.77%) of the studied NHL patients. Thrombosis was illustrated at initial lymphoma diagnosis in 46 (5.9%) patients, 61 (7.9%) patients experienced VTE while on chemotherapy. The median time from NHL diagnosis to thrombosis development was 8 months (range:1–96), Table 1. Types and frequencies of detected thrombosis are illustrated in Fig. 1.

### Thrombosis risk assessment scores

According to Khorana score results showed only 2 risk groups: 262/311 (84.2%) patients in the intermediate-risk group and 49/311 (15.8%) patients in the high-risk group. In the CONKA risk score, there were also 2 groups: low [*n* = 649, 83.5%] and high [*n* = 128, 16.5%]. While in ThroLy RAM, 121/311 (38.9%), 160/311 (51.4%), and 30/311 (9.6%) patients were considered low, intermediate, and high risk for VTE development, respectively Table 1.

### Comparison of patients’ characteristics among NHL patients with and without VTE at presentation

Forty-six NHL had VTE at presentation and before starting treatment. Those patients had significantly poorer PS, bulky lesions, mediastinal masses, and higher risk IPI scores (int-high and high) than lymphoma patients without VTE at initial diagnosis (37% vs. 19.2%, 58.7% vs. 29.1%, 19.6% vs. 3.1%, and 55.8% vs. 37.3% with ***P*** values 0.004, < 0.001, < 0.001 and 0.05, respectively). Patients with VTE had higher levels of LDH and higher incidence of hepatic cirrhosis, however, these did not illustrate significant statistical differences Table 2. Binary logistic regression analysis was conducted for the prediction of VTE before therapy in patients using PS, bulky lesions, and mediastinal mass. PS 2–4, the presence of either bulky lesion or mediastinal mass were independent predictors of VTE in univariate analysis and multivariate analysis Table 3.

**Table 2:** Comparison of patients’ characteristics among patients with and without thrombosis at presentation

Patients’ characteristics		Without VTE (*n* = 731)	With VTE (*n* = 46)	*P*-value
Age (years)	Median (Min-Max)	53.0 (18–81)	56.5 (23–98)	0.410
Gender	Male Female	370 (50.6%) 361 (49.4%)	29 (63.0%) 17 (37.0%)	0.102
Comorbidities (*n* = 725)	Negative HTN DM HTN/DM	500 (73.4%) 64 (9.4%) 74 (10.9%) 43 (6.3%)	33 (75.0%) 5 (11.4%) 3 (6.8%) 3 (6.8%)	0.841
Virology (*n* = 664)	Negative HCV HBV	303 (48.6%) 311 (49.9%) 9 (1.4%)	18 (43.9%) 22 (53.7%) 1 (2.4%)	0.764
Liver cirrhosis	Yes	65 (8.9%)	7 (15.2%)	0.151
B symptoms (*n* = 743)	Absent Present	461 (66.0%) 237 (34.0%)	28 (62.2%) 17 (37.8%)	0.600
PS	< 2 2–4	591 (80.8%) 140 (19.2%)	29 (63.0%) 17 (37.0%)	**0.004**
WBC (x 10^9^/L)	Median (Min-Max)	7.2 (1.2-133.8)	7.45 (3.0-23.6)	0.684
ANC ﻿(x 10^9^/L)	Median (Min-Max)	4.4 (0.3–23.5)	4.6 (1.3–21.4)	0.607
ALC ﻿(x 10^9^/L)	Median (Min-Max)	1.8 (0.1-114.3)	1.53 (0.18–5.6)	0.457
AMC ﻿(x 10^9^/L)	Median (Min-Max)	0.599 (0.06–6.06)	0.500(0.20–11.9)	0.816
NLR	Median (Min-Max)	2.45 (0.06-51.0)	2.53 (0.36–65.54)	0.583
MLR	Median (Min-Max)	0.307 (0.02-7.0)	0.322 (0.15–4.25)	0.268
HB (g/dl)	Median (Min-Max)	12.0 (3.5–17.2)	11.5 (4.3–15.1)	0.932
RDW (%)	Median (Min-Max)	15.2 (10-28)	15.3 (10-20)	0.876
Platelets (﻿x 10^9^/L)	Median (Min-Max)	226.0 (16–889)	237.5 (86–903)	0.948
MPV (fl)	Median (Min-Max)	9.1 (0.1–17.2)	8.8 (0.7–14.6)	0.471
PDW (fl)	Median (Min-Max)	17.94 (10.6–28.0)	18.3 (16.0-21.7)	0.537
PLR	Median (Min-Max)	125.5 (2.11–1340)	152.7 (24.1-1638.4)	0.515
Albumin (g/dl)	Median (Min-Max)	4.0 (1.47 − 5.9)	3.9 (1.7–5.4)	0.770
LDH (U/L)	Median (Min-Max)	411.0(100–2231)	455.0(186–4731)	0.067
Ki67 (*n* = 97)	Median (Min-Max)	70.0 (5–95)	70.0 (60–90)	0.616
Subtype	DLBCL Anaplastic T-cell rich NHL	674 (92.2%) 25 (3.4%) 32 (4.4%)	45 (97.8%) 1 (2.2%) 0 (0.0%)	0.307
Cell of origin (*n* = 222)	Non-germinal center Germinal center	90 (42.9%) 120 (57.1%)	8 (66.7%) 4 (33.3%)	0.138
IPI (*n* = 703)	Low Low-intermediate Intermediate-high High	162 (24.5%) 252 (38.2%) 174 (26.4%) 72 (10.9%)	5 (11.6%) 14 (32.6%) 15 (34.9%) 9 (20.9%)	0.051
Bulky lesion	Yes	213 (29.1%)	27 (58.7%)	**< 0**.**001**
Mediastinal mass	Yes	23 (3.1%)	9 (19.6%)	**< 0.001**
GIT	Yes	158 (21.6%)	9 (19.6%)	0.743
Thyroid	Yes	13 (1.8%)	1 (2.2%)	0.578
Renal	Yes	27 (3.7%)	4 (8.7%)	0.104
Reproductive system	Yes	20 (2.7%)	2 (4.3%)	0.379
Chest /CVS	Yes	55 (7.5%)	2 (4.3%)	0.569
Musculoskeletal	Yes	47 (6.4%)	3 (6.5%)	1.00
CNS	Yes	12 (1.6%)	0 (0.0%)	1.00
Bone marrow infiltration (n = 758)	Yes	83 (11.6%)	2 (4.4%)	0.218
Pharyngeal	Yes	75 (10.3%)	1 (2.2%)	0.076
Spleen	Yes	256 (35.0%)	16 (34.8%)	0.974

**Table 3:** Logistic regression for prediction of VTE at presentation

Independent predictors	Univariable			Multivariable		
	*P value*	OR	95% CI	*P* value	OR	95% CI
PS (2–4 vs. < 2)	0.005	2.475	1.323–4.630	0.022	2.137	1.113–4.101
Bulky lesion Yes	< 0.001	3.456	1.881–6.349	0.002	2.768	1.471–5.209
Mediastinal mass Yes	< 0.001	7.488	3.237–17.318	< 0.001	5.073	2.096–12.274

OR: odds ratio, CI: Confidence interval

### Comparison of patients’ characteristics among NHL patients with and without VTE on chemotherapy

VTE was documented in 61 NHL while on chemotherapy. Those patients had significantly poorer PS, bulky lesions, liver cirrhosis, and higher risk IPI scores (int-high and high) than those without VTE after chemotherapy (34.4% vs. 19%, 44.3% vs. 29.7%, 16.4% vs. 8.7%, and 54.6% vs. 37.1% with ***P*** values 0.004, 0.019, 0.046 and < 0.001, respectively). Patients on chemotherapy who developed VTE had significantly higher ANC, neutrophil/lymphocyte ratio (NLR), monocyte-to-lymphocyte ratio (MLR), platelet-lymphocyte ratio (PLR) ratio, and LDH levels, while they had significantly lower absolute lymphocytic counts (ALC), mean platelet volume (MPV) and serum albumin levels in comparison to NHL patients who did not have VTE Table 4. NHL lymphoma patients with VTE had significantly poorer responses, and higher relapse (*P* < 0.001, and 0.009, respectively).

**Table 4:** Comparison of patients’ characteristics among patients with and without thrombosis on chemotherapy

Patients’ characteristics		Without VTE (*n* = 716)	With VTE (*n* = 61)	*P*-value
Age (years)	Median (Min-Max)	53.0 (18–91)	55.0 (18–98)	0.736
Gender	Male Female	370 (51.7%) 346 (48.3%)	29 (47.5%) 32 (52.5%)	0.535
Comorbidities (*n* = 725)	Negative HTN DM HTN/DM	491 (73.5%) 64 (9.6%) 72 (10.8%) 41 (6.1%)	42 (73.6%) 5 (8.8%) 5 (8.8%) 5 (8.8%)	0.847
Virology (*n* = 664)	Negative HCV HBV	292 (47.8%) 309 (50.6%) 10 (1.6%)	29 (54.7%) 24 (45.3%) 0 (0.0%)	0.444
Liver cirrhosis	Yes	62 (8.7%)	10 (16.4%)	**0.046**
b symptoms (*n* = 743)	Absent Present	454 (66.3%) 231 (33.7%)	35 (60.3%) 23 (39.7%)	0.360
PS	< 2 2–4	580 (81.0%) 136 (19.0%)	40 (65.6%) 21 (34.4%)	**0.004**
WBC (﻿x 10^9^/L)	Median (Min-Max)	7.21 (1.2-133.8)	7.78 (1.9–20.5)	0.282
ANC ﻿(x 10^9^/L)	Median (Min-Max)	4.3 (0.3–23.5)	5.4 (1.0-14.5)	**0.002**
ALC ﻿(x 10^9^/L)	Median (Min-Max)	1.8 (0.1-114.3)	1.38 (0.18–2.8)	**< 0.001**
AMC ﻿(x 10^9^/L)	Median (Min-Max)	0.56 (0.06–6.06)	0.6 (0.1–11.9)	0.222
NLR	Median (Min-Max)	2.36 (0.06-51.0)	4.07 (0.81–65.54)	**< 0.001**
MLR	Median (Min-Max)	0.294 (0.02-7.0)	0.462 (0.04–4.25)	**< 0.001**
HB (g/dl)	Median (Min-Max)	12.10 (4.3–17.2)	11.5 (3.5–17.0)	0.185
RDW (%)	Median (Min-Max)	15.2 (10-28)	15.35 (11-28)	0.887
Platelets ﻿(x 10^9^/L)	Median (Min-Max)	224.0 (16.0-889)	239.0 (88.6–903.0)	0.379
MPV (fl)	Median (Min-Max)	9.2 (0.1–17.2)	8.0 (1.7–13.4)	**0.014**
PDW (fl)	Median (Min-Max)	17.9 (10.6–24.0)	18.1 (16.0–28.0)	0.473
PLR	Median (Min-Max)	123.5 (2.11–1340)	188.2 (47.1-1638.4)	**< 0.001**
Albumin (g/dl)	Median (Min-Max)	4.0 (1.47–5.90)	3.8 (2.0-5.3)	**0.013**
LDH (U/L)	Median (Min-Max)	404.5 (100–2231)	510.5(113–4731)	**0.022**
Ki67 (*n* = 97)	Median (Min-Max)	70.0 (5–95)	70.0 (15–90)	0.980
Subtype	DLBCL Anaplastic T-cell rich NHL	661 (92.3%) 23 (3.2%) 32 (4.5%)	58 (95.1%) 3 (4.9%) 0 (0.0%)	0.196
Cell of origin (*n* = 222)	Non-germinal center Germinal center	87 (43.5%) 113 (56.5%)	11 (50.0%) 11 (50.0%)	0.560
IPI (*n* = 703)	Low Low-intermediate Intermediate-high High	163 (25.2%) 245 (37.8%) 174 (26.9%) 66 (10.2%)	4 (7.3%) 21 (38.2%) 15 (27.3%) 15 (27.3%)	**< 0.001**
Bulky lesion	Yes	213 (29.7%)	27 (44.3%)	**0.019**
Mediastinal mass	Yes	27 (3.8%)	5 (8.2%)	0.095
GIT	Yes	156 (21.8%)	11 (18.0%)	0.493
Thyroid	Yes	13 (1.8%)	1 (1.6%)	1.00
Renal	Yes	29 (4.1%)	2 (3.3%)	1.00
Reproductive system	Yes	22 (3.1%)	0 (0.0%)	0.407
Chest /CVS	Yes	54 (7.5%)	3 (4.9%)	0.612
Musculoskeletal	Yes	44 (6.1%)	6 (9.8%)	0.271
CNS	Yes	9 (1.3%)	3 (4.9%)	0.061
Bone marrow infiltration (*n* = 758)	Yes	78 (11.2%)	7 (11.7%)	0.908
Pharyngeal	Yes	70 (9.8%)	6 (9.8%)	0.988
Spleen	Yes	253 (35.3%)	19 (31.1%)	0.510
Khorana risk score (*n* = 311)	Intermediate High	240 (85.1%) 42 (14.9%)	22 (75.9%) 7 (24.1%)	0.193
CONKO risk score	Low High	600 (83.8%) 116 (16.2%)	49 (80.3%) 12 (19.7%)	0.483
Throly risk score (*n* = 311)	Low Intermediate High	112 (39.7%) 143 (50.7%) 27 (9.6%)	9 (31.0%) 17 (58.6%) 3 (10.3%)	0.655
Anthracycline based chemotherapy	Non-anthracycline-based Anthracycline based	48 (6.7%) 668 (93.3%)	8 (13.1%) 53 (86.9%)	0.063
Response (*n* = 761)	CR PR SD PD	433 (62%) 141 (20.2%) 49 (7.0%) 76 (10.8%)	24 (38.7%) 15 (24.2%) 5 (8.1%) 18 (29%)	**< 0.001**
Relapse (*n* = 457)	Non relapsed Relapsed	335 (77.4%) 98 (22.6%)	13 (54.2%) 11 (45.8%)	**0.009**

### Predictors of VTE in NHL patients

Cox regression analysis was conducted for the prediction of VTE after chemotherapy in NHL patients using PS, laboratory parameters, bulky lesions, liver cirrhosis, IPI, treatment response, and relapse status. Higher PS [2–4], ANC, NLR, MLR, PLR, and LDH levels, presence of a bulky lesion, higher IPI, partial response (PR), progressive disease (PD), and relapse were independent predictors of VTE, while elevated ALC and elevated albumin were significant protective of VTE in univariate analysis. In multivariate analysis, only higher PS, ANC, NLR, PLR, and LDH were independent predictive factors for the VTE Table 5.

**Table 5:** Cox regression for prediction of VTE on chemotherapy

Independent predictors	Univariable			Multivariable		
	*P value*	HR	95% CI	*P* value	HR	95% CI
PS (2–4 vs. < 2)	**0.004**	**2.178**	**1.285–3.694**	**0.003**	**6.069**	**1.881–19.582**
ANC ﻿(x 10^9^/L)	**< 0.001**	**1.128**	**1.059–1.202**	**0.034**	**1.256**	**1.018–1.549**
ALC ﻿(x 10^9^/L)	**< 0.001**	**0.479**	**0.340–0.674**	0.540	0.775	0.343–1.752
NLR	**< 0.001**	**1.061**	**1.039–1.083**	**0.049**	**1.150**	**1.105–1.307**
MLR	**0.001**	**1.564**	**1.277–1.915**	0.353	2.043	0.452–9.224
MPV(fl)	0.133	0.929	0.844–1.023			
PLR	**< 0.001**	**1.002**	**1.001–1.003**	**0.010**	**1.005**	**1.001–1.009**
Albumin (g/dl)	**0.002**	**0.565**	**0.391–0.817**	0.724	0.869	0.399–1.895
LDH (U/L)	**< 0.001**	**1.002**	**1.001–1.004**	**0.007**	**1.002**	**1.001–1.003**
Bulky lesion Yes	**0.019**	**1.828**	**1.103–3.030**	0.260	1.896	0.624–5.764
Liver cirrhosis Yes	0.061	1.912	0.971–3.766			
**IPI** Low-intermediate Intermediate-high High	**0.025** **0.029** **< 0.001**	**3.400** **3.408** **8.413**	**1.167–9.904** **1.131–10.270** **2.792–25.35**	0.680 0.587 0.845	1.432 1.576 1.108	0.259–7.921 0.879–4.219 0.195–6.869
**Response** PR SD PD	**0.034** 0.169 **< 0.001**	**2.033** 1.976 **4.294**	**1.055–3.920** 0.748–5.219 **2.303–8.008**	0.152 ---- 0.130	2.008 ---- 2.365	0.993–4.062 ---------- 0.986–5.151
**Relapse**	**0.014**	**2.724**	**1.220–6.081**	0.333	1.711	0.576–5.082

HR: Hazard ratio, CI: Confidence interval

**Fig. 2:**
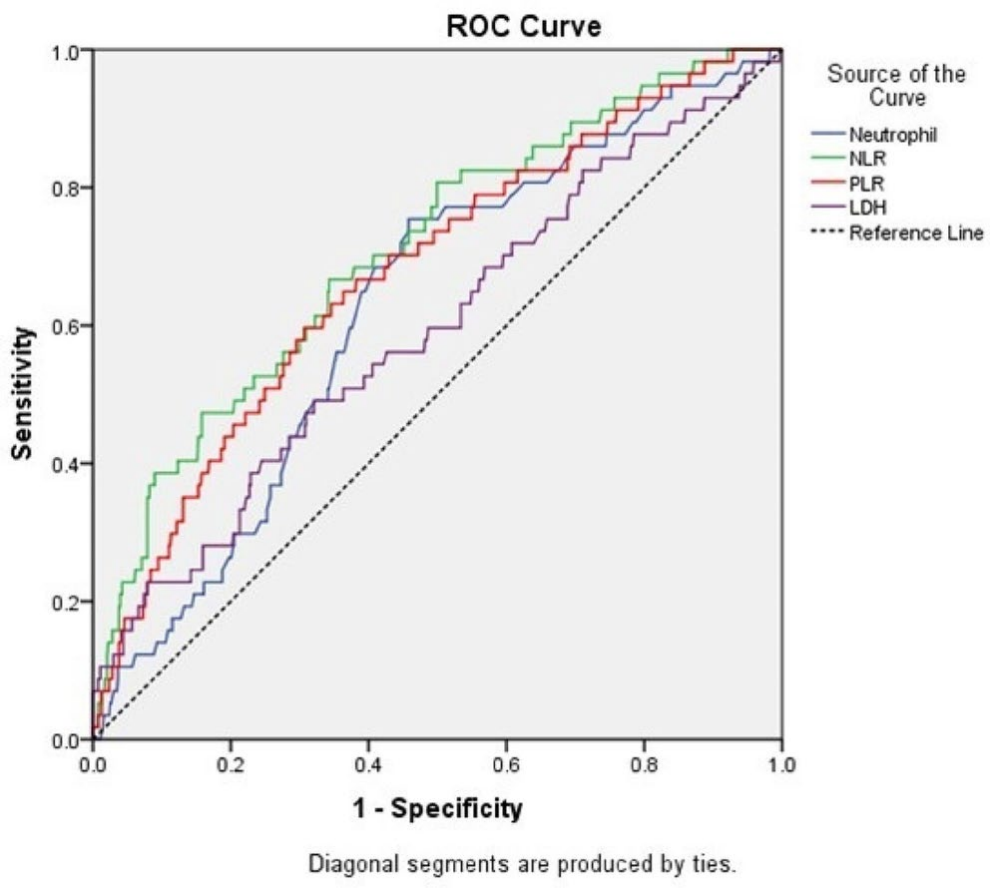
Receiver operating characteristic (ROC) plot of ANC, NLR, PLR, and LDH predicting the presence of VTE. The area under the curve was  0.628,  0.708,  0.676 and 0.592, respectively

ROC curves of different variables were performed in search for predictors of thrombosis in lymphoma patients. ROC curve showed cut-off values of 4.875 × 10^9^/L, 2.985, 144.85, and 417.5 U/L for ANC, NLR, PLR, and LDH, respectively with a good AUC and acceptable sensitivity and specificity and significant *P*-values Table 6, therefore, these parameters could serve as potential biomarkers for prediction of thrombosis in NHL Fig. 2.

**Table 6:** Performance characteristics of ANC, NLR, PLR, and LDH for discrimination between patients with and without VTE on chemotherapy

	ANC	NLR	PLR	LDH
AUC	0.628	0.708	0.676	0.592
*P*	0.001	< 0.001	< 0.001	0.021
95% CI	0.557–0.698	0.637–0.780	0.603–0.750	0.511–0.673
Cut off	4.875	2.985	144.85	417.5
Sensitivity (%)	68.4%	68.4%	66.7%	59.6%
Specificity (%)	59.1%	62.1%	61.8%	51.3%

**Fig. 3:**
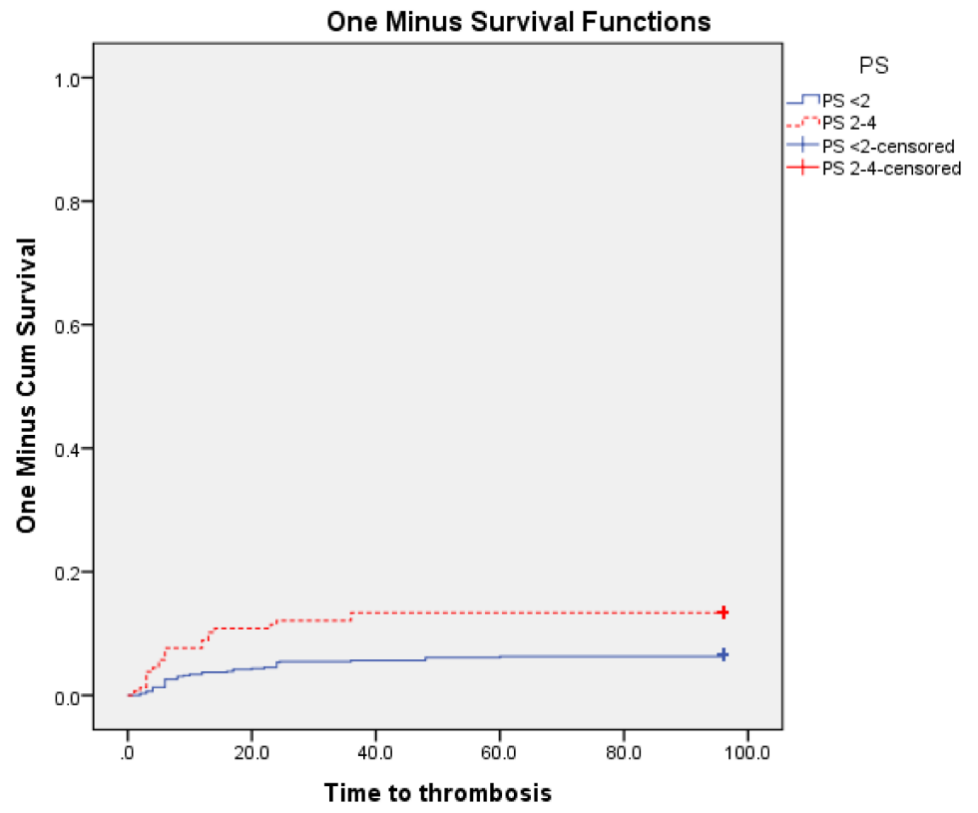
Kaplan-Meier’s curves for VTE according to PS groups. VTE were significantly higher in PS 2–4 group (13.4% at 96 months interval) than PS < 2 group (6.5% at 96 months interval) (P 0.003)

**Fig. 4:**
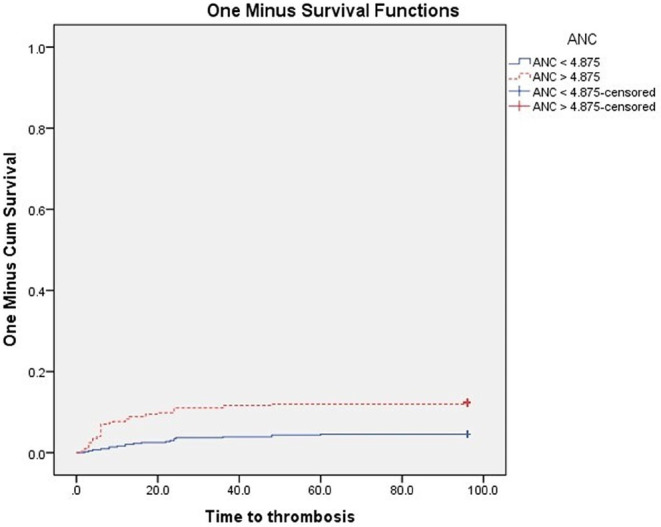
Kaplan-Meier’s curves for VTE according to ANC groups. VTE were significantly higher in ANC > 4.875 × 10^9^/L group (12.3% at 96 months interval) than ANC < 4.875 × 10^9^/L group (4.6% at 96 months interval) (*P* < 0.001)

**Fig. 5:**
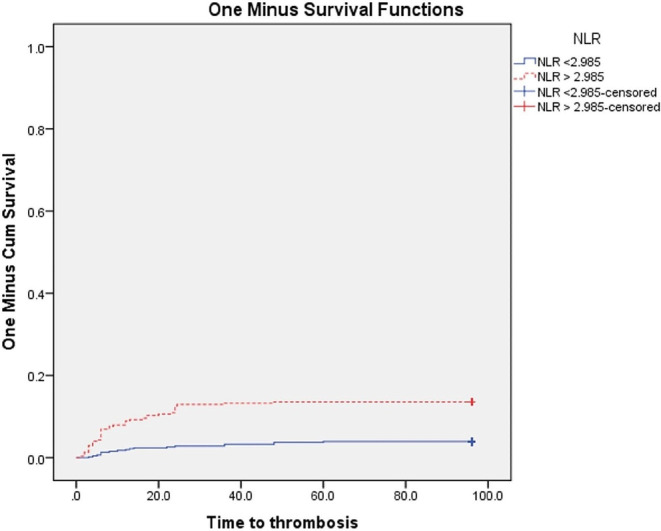
Kaplan-Meier’s curves for VTE according to NLR groups. VTE were significantly higher in NLR > 2.985 group (13.6% at 96 months interval) than NLR < 2.985 group (3.9% at 96 months interval) (*P* < 0.001)

**Fig. 6:**
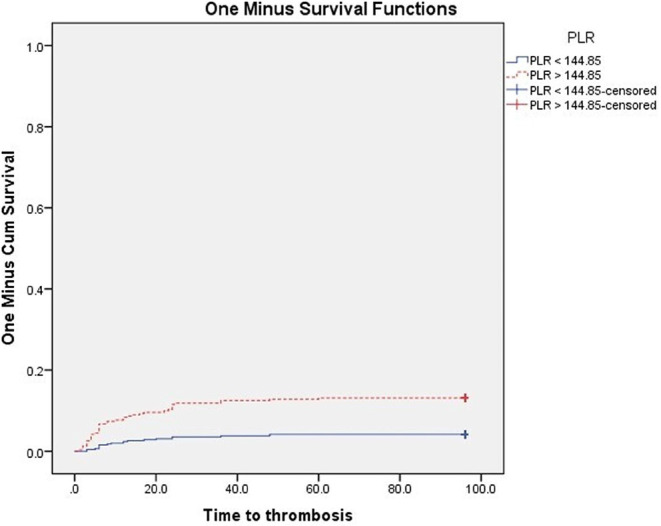
Kaplan-Meier’s curves for VTE according to PLR groups. VTE were significantly higher in PLR > 144.85 group (13.1% at 96 months interval) than PLR < 144.85 group (4.2% at 96 months interval) (*P* < 0.001)

**Fig. 7:**
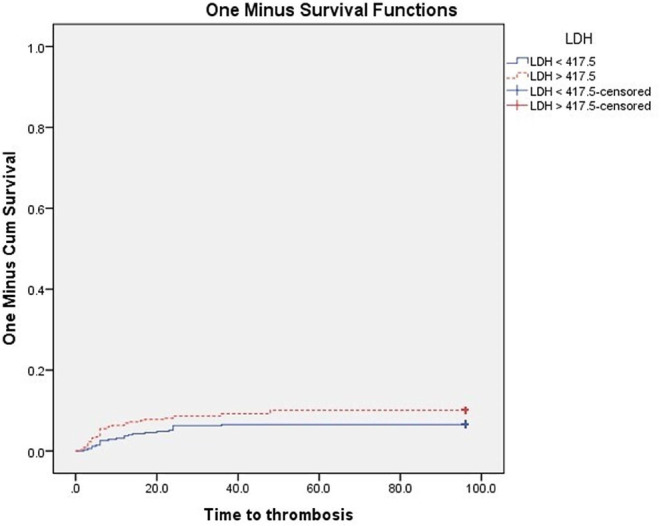
Kaplan-Meier’s curves for VTE according to LDH groups. VTE were higher in LDH > 417.5 U/L group (10.1% at 96 months interval) than LDH < 417.5 U/L group (6.5% at 96 months interval) without statistical significance (P 0.087)

At the end of the follow-up, VTE were significantly higher in patients with PS ≥ 2 (P 0.003), and in patients with ANC, NLR, PLR above the cut-off values subgroups vs. below cut-off values subgroups (*P* < 0.001 for each subgroup) Figs. 3, 4, 5 and 6. Also VTE were higher in LDH > 417.5 U/L group vs. LDH < 417.5 U/L group but without statistical significance (P 0.087) Fig. 7.

### Survival analysis

**Fig. 8:**
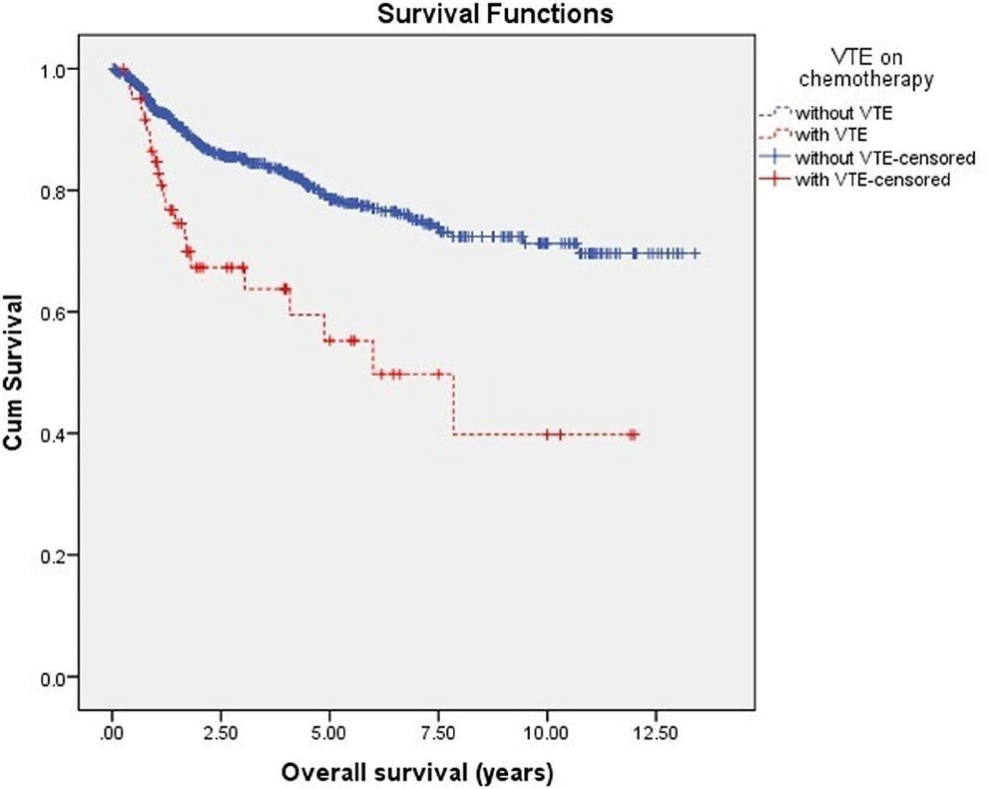
OS of patients with and without VTE on chemotherapy using Kaplan-Meier analysis (*P* <0.001)

The median follow-up was 3.14 years (range 0.02–13.4 years). The multivariate analysis identified thrombosis developed while on chemotherapy, hypoalbuminemia, higher IPI scores, and refractory/relapsed NHL as independent predictors for dismal OS Fig. 8; Table 7.

**Table 7:** Cox regression for prediction of OS:

Independent predictors	Univariable			Multivariable		
	*P value*	HR	95% CI	*P* value	HR	95% CI
Age (years)	0.422	1.005	0.993–1.017			
Gender	0.066	0.738	0.534–1.020			
Thrombosis at presentation	0.421	1.275	0.706–2.302			
Thrombosis on chemotherapy	**< 0.001**	**2.579**	**1.638–4.060**	**0.012**	**1.946**	**1.154–3.281**
B symptoms	0.273	1.210	0.861–1.701			
WBC ﻿(x 10^9^/L)	0.605	1.091	0.959–1.137			
ANC ﻿(x 10^9^/L)	0.931	1.001	0.946–1.052			
ALC ﻿(x 10^9^/L)	0.180	0.925	0.825–1.037			
AMC ﻿(x 10^9^/L)	0.431	1.109	0.858–1.433			
NLR	0.064	1.027	0.998–1.056			
MLR	0.072	1.420	0.855–1.776			
HB (g/dl)	**< 0.001**	**0.879**	**0.819–0.944**	0.257	0.947	0.863–1.040
RDW (%)	0.074	1.047	0.996–1.101			
Platelets ﻿(x 10^9^/L)	0.558	1.001	0.998–1.003			
MPV (fl)	0.910	0.976	0.938–1.075			
PDW (fl)	0.341	1.043	0.956–1.138			
PLR	0.195	1.001	0.999–1.003			
Albumin (g/dl)	**< 0.001**	**0.514**	**0.400–0.660**	**0.008**	**0.655**	**0.479–0.896**
Bulky lesion	0.092	1.330	0.954–1.854			
**IPI** Low-intermediate Intermediate-high High	**< 0.001** **< 0.001** **< 0.001**	**3.355** **4.333** **7.715**	**1.700-6.625** **2.174–8.638** **3.443–14.951**	0.086 **0.034** **0.018**	1.906 **2.239** **2.704**	0.914–3.975 **1.063–4.716** **1.183–6.184**
**Response** PR SD PD	**< 0.001** **< 0.001** **< 0.001**	**3.114** **5.760** **12.469**	**1.978–4.902** **3.399–9.761** **8.282–18.772**	**0.001** **< 0.001** **< 0.001**	**2.464** **5.584** **9.862**	**1.456–4.168** **2.973–10.490** **6.082–15.992**
**Relapse**	**< 0.001**	**12.227**	**6.048–24.716**	**< 0.001**	**11.801**	**5.014–27.778**

HR: Hazard ratio, CI: Confidence interval

## Discussion

In our study, the rate of VTE in NHL patients at diagnosis and on chemotherapy was 5.9 and 7.9%, respectively. Poorer PS, presence of bulky and mediastinal lesions were shown to be independent predictive factors for VTE at diagnosis. The 3 RAM used were not proven to be positive predictive scores, while the inflammatory markers: ANC, NLR, and PLR were found to be independent prognostic factors for VTE development in NHL patients on chemotherapy. Additionally, VTE on chemotherapy was an independent poor prognostic factor for OS.

In our study, 46 (5.9%) patients had documented thrombosis at their initial presentation. A meta-analysis has shown a 3.8% incidence of VTE at diagnosis before chemotherapy [24], while other studies recorded much higher frequencies ranging from 20–66% [19, 25]. We found that ECOG PS 2–4, the presence of either bulky lesion or mediastinal mass before chemoimmunotherapy initiation were identified as prognostic factors for VTE development in patients with NHL in both univariate and multivariate analysis.

Our data showed that the rate of VTE development was 7.9% in NHL patients after starting treatment. In a meta-analysis study, the overall incidence of VTE in lymphoma was 6.4% showing a higher incidence among NHL than HL patients [24]. Previous studies have shown that the risk for the development of VTE in DLBCL was 11% [26, 27]. In another study, the incidence of VTE in lymphoma was 9.8% [15]. These variable rates between published studies could be attributed to the different study designs and types of lymphoma included. We documented that intermediate-high/high IPI scores, PS 2–4, presence of bulky lesions were independent predictors of VTE on chemotherapy in univariate analysis. However, in multivariate analysis only higher PS was an independent predictive factor for VTE. The same observation by Otasevic et al. [15] was seen in our study that advanced-stage NHL had more frequent VTE, however, this did not show statistical significance. Similar findings were found by other studies [24, 28, 29].

Our data showed that the incidence of VTE in patients with liver cirrhosis was significantly higher in the NHL patients who developed VTE on chemotherapy than in those without VTE (***P*** 0.046). Cirrhotic patients present per se a higher risk of developing thrombotic complications [30]. A meta-analysis by Pasquale Ambrosino, confirmed the association between cirrhosis and VTE, patients with cirrhosis were at a higher risk of VTE than patients without cirrhosis (3.7% vs. 1.8%; odds ratio [OR] = 1.703; 95% CI 1.333–2.175; *P* < 0.0001), which amounted to an attributable risk, defined as the risk of VTE among control patients divided by the risk for VTE among cirrhotic patients, of 51.3% [31].

No significant differences regarding gender, age, Hb concentration, WBC, or platelet counts were found between NHL with or without thrombosis on chemotherapy, while a previous study conducted on a larger sample size of both NHL and HL considered male gender, older age, and elevated platelet counts as independent factors of VTE in lymphoma. However, like ours, other studies showed no significant difference between different chemo-immunotherapies used as regards thrombosis risk [10, 32]. They suggested that the risk of VTE may be more related to the histologic type of NHL, as majority of the cases receiving anthracycline were DLBCL which constituted the larger percentage of the included lymphomas, rather than the type of chemotherapy [10].

High LDH and Low albumin levels were considered risk factors for VTE development in previous studies [19]. LDH plays an important role in various neoplasms’ development and progression, and it is considered a marker of cell destruction and tumor burden, therefore it is implicated in prognostic models of lymphomas [33]. Albumin inversely correlates with IL-6 levels and therefore it is considered an inflammatory marker [34]and elevated IL-6 levels are associated with increased VTE [35].

Risk assessment models (RAM) were developed to identify cancer patients at higher risk of developing VTE. The best-validated tool is the Khorana score [13]; various modifications have been tried over the years to improve its performance [21, 36–38]. Assessment of Khorana score validity in lymphoma patients by different studies have reached controversial results, some succeeded [39] while others similar to our data failed to demonstrate its efficacy to stratify patients at risk of VTE [37, 40–42]. Despite that CONKO score was a modified version of Khorana RAM, it did not show much improvement as regards discrimination potential in our studied patients and it lacked accuracy in identifying patients in need of thromboprophylaxis based on other studies results [37, 43]. Lastly, ThroLy score was an inaccurate model for VTE prediction in our cohort and in a previous study including DLBCL and HL patients [44], yet, it was a discriminator in Antic D et al. study which enrolled various subtypes of lymphoma from low-grade lymphomas who have a lower risk for VTE to aggressive lymphomas with higher risks for VTE [20] and Hikmat et al. [42] showed that DLBCL patients with high ThroLy scores had a significantly high risk for VTE development, they attributed their results to the aggressive nature of DLBCL and they modified the score by removing neutropenia from the variables.

Our univariate, multivariate, and ROC curve statistical analysis concluded that ANC, NLR, and PLR could serve as predictor factors of VTE in NHL. These are similar to findings detected in a recent study; their ROC curve concluded that NLR and PLR were predictors of VTE in lymphoma patients and NLR was considered an independent risk factor for VTE in lymphoma by their multivariate analysis [15]. A study conducted by Romano et al. considered NLR as an independent survival predictor in lymphoma and an indicator of immune response to malignancy [45]. In contrast, a previous study did not find a relation between NLR and PLR and the risk of VTE development in lymphoma patients [46]. PLR is an inflammatory marker and immune response indicator and is considered a prognostic factor in variable neoplasms. Elevated ANC, NLR, and PLR in DLBCL patients are assumed to be associated with higher infiltration index and tumor burden leading to poor prognosis in those patients, also high PLR could be secondary to increased platelet counts due to immune system dysfunction or decreased lymphocytes leading to accelerated tumor development and progression. A recent study showed that elevated PLR has a poor prognostic impact on lymphoma patients [47].

A previous study illustrated that lower ALC and MLR were associated with poor response and survival outcomes and low MLR was considered an independent poor prognostic factor [48]. Similar studies showed that lower MPV in lymphoma [16, 17] and hematological malignancies carries an increased risk of VTE development [49]. MPV represents the platelet volume and is a marker of platelet activation and an inflammatory marker as it declines due to platelet consumption in inflammatory situations. Lower MPV in hematological malignancies is a poor prognostic factor [50].

SEER database has proposed that survival trends in NHL will continue to improve through 2019 to 2023, survival rates were better among younger patients, limited Ann Arbor stages, female gender, and extranodal involvement [51]. Age and gender did not show survival differences among the studied patients in our study. VTE in lymphoma patients affects their quality of life and decreases survival rates [32]. Our analysis showed that the occurrence of thrombosis in NHL while on chemotherapy is an independent poor prognostic factor for OS. VTE was estimated to be one of the most common comorbidities in a large cohort of NHL and thus was part of the proposed 3-factor risk estimate scale (TRES), higher scores were associated with higher lymphoma-specific mortality [52].

## Conclusion

This study highlights the complexity of predicting VTE risk and the need for multifaceted approaches in clinical assessments. We suggest that inflammatory markers could serve as predictors of thrombotic events development in NHL, especially DLBCL. Higher NLR, PLR, ANC, and advanced PS were considered independent predictive factors for VTE. Further studies are needed to confirm the predictive value of inflammatory markers and to be incorporated in prognostic scoring models to help assign patients at high risk for VTE and initiate thromboprophylaxis for better quality of life for our patients, as development of VTE during therapy has shown to be an independent risk factor for dismal survival.

A large discrepancy is seen between studies in the assessment of the value of RAM as predictive scores for thrombosis. RAM has shown inferior performance across many studies in their ability to discriminate between low and high-risk patients for VTE; patients in low-risk groups have recorded thrombotic events, so further improvements are needed to enhance these scores.

Limitations to our study were the retrospective nature of the study, and few data were missing, however, we consider our sample size to be sufficient as we focused on aggressive NHL especially DLBCL, giving homogeneity to the study nature. We recommend conducting research on a larger sample size including other subtypes of NHL and even HL, multi-center, and prospective studies, to identify risk factors for thrombosis development in lymphoma.

## Data Availability

No datasets were generated or analysed during the current study.
